# Azithromycin attenuates airway inflammation in a mouse model of viral bronchiolitis

**DOI:** 10.1186/1465-9921-11-90

**Published:** 2010-06-30

**Authors:** Avraham Beigelman, Cassandra L Mikols, Sean P Gunsten, Carolyn L Cannon, Steven L Brody, Michael J Walter

**Affiliations:** 1 Division of Allergy, Immunology & Pulmonary Medicine, Department of Pediatrics, Washington University School of Medicine, St. Louis, MO; USA; 2 Division of Pulmonary and Critical Care Medicine, Department of Internal Medicine, Washington University School of Medicine, St. Louis, MO; USA

## Abstract

**Background:**

Viral bronchiolitis is the leading cause of hospitalization in young infants. It is associated with the development of childhood asthma and contributes to morbidity and mortality in the elderly. Currently no therapies effectively attenuate inflammation during the acute viral infection, or prevent the risk of post-viral asthma. We hypothesized that early treatment of a paramyxoviral bronchiolitis with azithromycin would attenuate acute and chronic airway inflammation.

**Methods:**

Mice were inoculated with parainfluenza type 1, Sendai Virus (SeV), and treated daily with PBS or azithromycin for 7 days post-inoculation. On day 8 and 21 we assessed airway inflammation in lung tissue, and quantified immune cells and inflammatory mediators in bronchoalveolar lavage (BAL).

**Results:**

Compared to treatment with PBS, azithromycin significantly attenuated post-viral weight loss. During the peak of acute inflammation (day 8), azithromycin decreased total leukocyte accumulation in the lung tissue and BAL, with the largest fold-reduction in BAL neutrophils. This decreased inflammation was independent of changes in viral load. Azithromycin significantly attenuated the concentration of BAL inflammatory mediators and enhanced resolution of chronic airway inflammation evident by decreased BAL inflammatory mediators on day 21.

**Conclusions:**

In this mouse model of paramyxoviral bronchiolitis, azithromycin attenuated acute and chronic airway inflammation. These findings demonstrate anti-inflammatory effects of azithromycin that are not related to anti-viral activity. Our findings support the rationale for future prospective randomized clinical trials that will evaluate the effects of macrolides on acute viral bronchiolitis and their long-term consequences.

## Background

Viral bronchiolitis is the most common acute infection of the lower respiratory tract in infancy, and is most often caused by the paramyxoviruses, especially respiratory syncytial virus (RSV). RSV will infect 95% of children by the age of 2 [1], and up to 3% of infected children will develop a severe bronchiolitis requiring hospitalization [2]. The rate of admissions has doubled in the past 2 decades [3]; as a result, severe RSV bronchiolitis is now the leading cause of hospitalization in infants younger the age of 1 year [4]. Chronic respiratory symptoms are common after severe RSV bronchiolitis, with about 40% of hospitalized children eventually developing asthma [5-8]. The development of asthma following RSV infection appears to be related to the severity of the initial infection [9]. RSV infection is not limited to children and contributes significantly to morbidity and mortality in the elderly population [10]. These findings suggest that attenuating the acute viral infection may be an effective strategy to attenuate the acute and long-term consequences of viral bronchiolitis.

No specific therapies are currently recommended for severe RSV bronchiolitis [11]. Ideally a beneficial pharmacologic agent would reduce acute morbidity as well as modify the anti-viral host response to avert the subsequent development of asthma. One class of potentially useful therapeutic agents is the macrolide antibiotics, since they possess distinct anti-inflammatory properties in addition to their antimicrobial effects [12-25]. Two clinical studies have evaluated macrolide treatment during severe RSV bronchiolitis in children, but these studies have yielded conflicting results [26,27]. Therefore, definitive conclusions regarding the usefulness of macrolides as a treatment of viral-bronchiolitis cannot be made at this time.

To examine the anti-inflammatory properties of macrolides in a high fidelity animal model of human RSV bronchiolitis, we tested the ability of azithromycin to modulate a well-characterized viral bronchiolitis model using a mouse parainfluenza type I virus, referred to as Sendai virus (SeV) [28,29]. SeV replicates at high efficiency in the mouse lung and results in an acute viral bronchiolitis, and chronic airway inflammation that persists for at least one year following viral inoculation [28,29]. We hypothesized that treatment of mouse SeV bronchiolitis with azithromycin during the acute infection would attenuate early airway inflammation, and also decrease the chronic post-viral pathologic abnormalities, such as immune cell accumulation and the mediators that drive these processes.

## Materials and methods

### Mice

C57BL/6J female mice were purchased from the Jackson Laboratory (Bar Harbor, ME). All mice were bred and housed under specific pathogen-free conditions at Washington University School of Medicine where sentinel mice (pathogen free ICN-strain) exhibited no serologic or histologic evidence of exposure to 15 murine pathogens (including SeV). Before performing these in vivo experiments, we investigated whether our colony of mice were actively infected or colonized with bacteria in the trachea and lungs. We obtained tracheal swabs and tissue samples from both lungs, from 5 mice, and plated them on tryptic soy agar plates supplemented with 5% sheep blood, incubated for 48 hours at 37°C and no colonies were identified. To determine if our colony of mice had serologic evidence of prior *Mycoplasma pulmonis *exposure, serum was collected for indirect ELISA using *Mycoplasma pulmonis *antigen-coated plates according to manufacturer's recommendations (Charles River Laboratories, Wilmington, MA). These results were negative for infection as previously included in our prior manuscript [30]. In addition, we have tested our SeV stock for bacterial contamination by streaking the viral stock on tryptic soy agar plates supplemented with 5% sheep blood followed by incubated for 48 hours at 37°C. No colonies were identified. The Institutional Animal Use and Care Committee of Washington University School of Medicine approved all animal experiments.

### Induction of viral bronchiolitis

**Figure 1:**
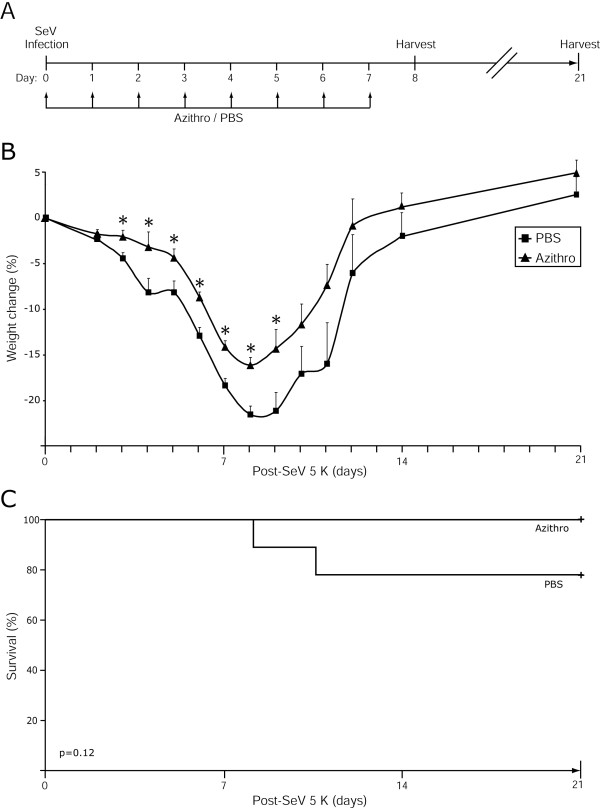
**Azithromycin attenuated viral-dependent weight loss**. **(A) **Experiment time line. Seven-week-old C57BL/6J female mice were inoculated with Sendai virus 5,000 egg infectious dose 50% (SeV 5 K). Mice were treated daily with PBS or azithromycin day 0 (one hour after SeV inoculation) through day 7. On days 8 and 21, bronchoalveolar lavage (BAL) fluid and lungs and were harvested. **(B) **Percentage of weight change from baseline (day 0) in PBS (black square) versus azithromycin (black triangle) treated mice. Values are the mean ± SEM (n = 23 in each group). A significant decrease between PBS and azithromycin treatment is indicated (*, p < 0.05). **(C) **Kaplan-Meier analysis of survival. No statistical difference between treatment groups (n = 23 in each group) was determined by log-rank test.

SeV, a mouse parainfluenza type 1 virus that is similar to the human paramyxoviruses (a class of viruses that includes RSV, metapneumovirus, and parainfluenza viruses) was used to generate airway inflammation of the small airways (i.e., viral bronchiolitis) as we previously described [29,31,32]. On day zero, seven-week-old C57BL/6J female mice underwent anesthesia and intranasal inoculation with SeV (Fushimi Strain, ATCC #VR-105) at 5,000 egg infectious dose 50% (5 K). This dose generates a sub-lethal tracheobronchitis/bronchiolitis viral infection [31,32]. On days 8 and 21 post-inoculation, the mice were anesthetized and euthanized for bronchoalveolar lavage (BAL) fluid and lung tissue collection as we previously described (see experiment design, Figure 1A) [29-33]. The day 8 time point corresponds to the peak of acute airway inflammation, while the day 21 time point corresponds to a chronic inflammatory phase of the infection [29,31]. Each experiment was repeated 3 times with multiple animals in each treatment group.

### Azithromycin treatment

Mice were treated daily with subcutaneous azithromycin (50 mg/kg dissolved in 100 μL sterile PBS, purchased from Pfizer Pharmaceuticals, Dublin, Ireland), from day 0 (one hour after SeV inoculation) through day 7 post-viral inoculation. Mice that were treated with subcutaneous 100 μL sterile PBS served as a control for the azithromycin treatment. Azithromycin dose was determined based on our previous pharmacokinetic studies [30] which demonstrated that daily subcutaneous treatment of mice with azithromycin (50 mg/kg) produced serum levels similar to those observed in humans treated with the recommended azithromycin dose. Overall, a higher dosage of azithromycin is required in mice than humans due to more rapid liver metabolism in mice, resulting in an elimination half-life of 2.3 hours compared to 68 hours in humans [34,35].

### Mouse specimen analyses

BAL and lung tissue harvest were performed as previously described [29-32]. Two blinded observers determined the BAL immune cell differential using standard light microscopy criteria as described previously [30,31]. BAL inflammatory mediators were analyzed using a multiplex flow-cytometry based assay according to manufacturer's recommendations (Bio-Rad Laboratories) and as previously described [30,31]. The detection limit for the Bio-plex mouse cytokine 23-plex panel (Bio-Rad) is: IL-1α - 2 pg/ml; IL-1β - 2 pg/ml; IL-2 - 3 pg/ml; IL-3 - 2 pg/ml; IL-4 - 3 pg/ml; IL-5 - 2 pg/ml; IL-6 - 2 pg/ml; IL-9 - 15 pg/ml; IL-10 - 2 pg/ml; IL-12 (p40) - 2 pg/ml; IL-12 (p70) - 4 pg/ml; IL-13 - 9 pg/ml; IL-17 - 1 pg/ml; eotaxin - 148 pg/ml; G-CSF - 1 pg/ml; GM-CSF - 7 pg/ml; IFN-γ - 6 pg/ml; KC - 3 pg/ml; CCL2/JE - 14 pg/ml; CCL3/MIP-1β - 24 pg/ml; CCL4/MIP-1ί - 2 pg/ml; CCL5/RANTES - 5 pg/ml; and TNF-α - 6 pg/ml.

Lung sections were stained with hematoxylin and eosin (H&E) and Periodic Acid-Schiff (PAS) [30,31]. Quantification of mucus producing cells was performed by counting the number of airway cells that stained with PAS positive cells and using a PAS score as previously described [30,36,37]. Peripheral blood leukocyte counts were performed using an automated veterinary hematologic analyzer with a pre-programmed murine calibration mode (Hemavet 950FS, Drew Scientific, Waterbury, CT) as previously described [31].

### PCR Quantification of Sendai virus

The quantity of Sendai virus-specific RNA was determined from whole lung using a TaqMan one-step fluorogenic RT-PCR reaction according to the manufacturer's recommendation (Applied Biosystems, Foster City, CA) [29,31]. Lung tissue was placed in RNA Later (Applied Biosystems), homogenized with stainless steel beads for 3 min (Biospect Products Inc., Bartlesville, OK), and column purified with an RNeasy mini kit according to the manufacturer's recommendations (Qiagen, Alencia, CA). Duplicate serial 10-fold dilutions of total RNA from Sendai-infected lung tissue underwent one-step fluorogenic RT-PCR for detection of Sendai virus nucleocapsid protein transcripts (upstream primer 5'-TCCACCCTGAGGAGCAGG-3'; downstream primer 5'-ACCCGGCCATCGTGAACT-3'; probe 5'-6FAM-TGGCAGCAAAGCAAAGGGTCTGGA-TAMRA-30) and murine GAPDH specific RNA (proprietary primer/probe combination, Applied Biosystems; #MM99999915G1) to construct standard curves. Sendai values were calculated as the mean of duplicate samples from reactions with a cycle threshold between 20 and 25 and final results were normalized to GAPDH and reported as the Sendai/GAPDH ratio.

### Statistical analysis

Means from multiple groups (BAL cell counts and inflammatory mediator concentrations on day 8) were analyzed for statistical significance using a one-way analysis of variance (ANOVA) and post hoc comparison to identify significant differences between specific groups. An independent group's t-test was used to compare means from two groups (BAL cell counts and inflammatory mediator concentrations on day 21). The Mann-Whitney test was used to compare the lung PAS scores (an ordinal variable). The significance level for all tests was 0.05. Data were analyzed using SPSS 15 software (Chicago, IL).

## Results

### Azithromycin attenuated viral-dependent weight loss

To determine if azithromycin could confer anti-inflammatory properties in a mouse model of viral bronchiolitis, we inoculated mice on day 0 with SeV (5,000 egg infectious dose 50%, 5 K) and treated the mice daily with azithromycin or PBS from day 0 to day 7 (Figure 1A). Mice treated with azithromycin had attenuated weight loss compared to PBS treated mice, with significant differences observed on days 3 through 9 post-inoculation (Figure 1B). We noted a trend toward lower mortality in the azithromycin treated mice (Figure 1C): 23/23 mice survived in the azithromycin group versus 21/23 in the control group (p = 0.12). To assess whether azithromycin treatment would be beneficial using a higher infectious dose, we performed an experiment using a lethal infectious dose of virus (50,000 egg infectious dose 50%, 50 K). Treatment of this lethal infection with azithromycin did not significantly alter weight loss, overall survival or delay the time of death compared to treatment with PBS (N = 5 in each cohort). These results demonstrated that azithromycin treatment, in the 5 K infectious dose, attenuated some clinical features of the severity of the acute viral bronchiolitis. Therefore, we next sought to characterize the impact of azithromycin treatment on lung inflammation.

### Azithromycin attenuated viral-dependent airway inflammation

**Figure 2:**
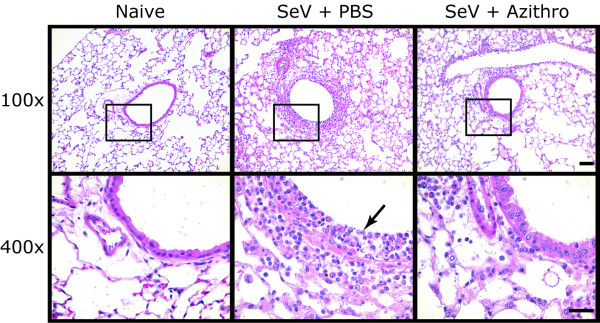
**Azithromycin attenuated viral-dependent airway inflammation**. Mice were inoculated with SeV and treated as in Figure 1. Eight days post-viral inoculation, lung sections were obtained from naive mice (Naive, left column); SeV infected mice treated with PBS (SeV + PBS, middle column), and SeV infected mice treated with azithromycin (SeV + Azithro, right column). Representative photomicrographs of hematoxylin and eosin stained lung sections are shown (n = 11, Azithro; n = 12, PBS). Inflammatory cells within the airway are indicated (arrow). Bar = 50 μm (top) and 25 μm (bottom).

**Figure 3:**
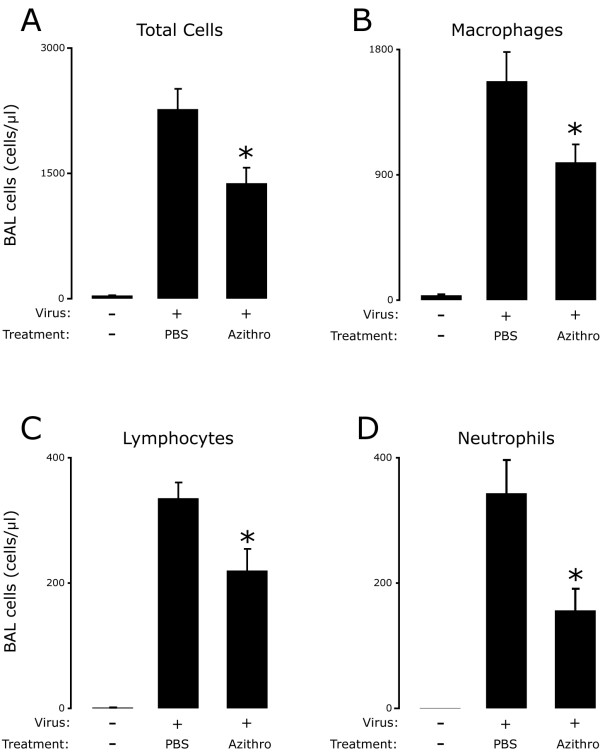
**Azithromycin attenuated viral-dependent accumulation of total cells, macrophages, lymphocytes and neutrophils in the BAL**. BAL from mice treated as in Figure 1 was analyzed for total and differential cell number eight days post-inoculation. Groups are labeled as in Figure 2A and values are the mean ± SEM (n = 11, Azithro; n = 12, PBS) of total BAL cells (**A**), macrophages (**B**), lymphocytes (**C**) and neutrophils (**D**). A significant decrease between PBS and azithromycin treatment is indicated (*, p < 0.05).

To determine whether azithromycin treatment could attenuate airway inflammation during the SeV 5 K infection, we examined the lungs from PBS and azithromycin treated mice 8 days following viral inoculation. This time point correlates to peak post-viral airway inflammation [31]. Compared to naive mice, SeV inoculated mice treated with PBS had an accumulation of immune cells predominantly in the peribronchial space, in the airway lumen, and to a lesser extent in the alveolar spaces. In some airways we observed severe epithelial cell injury with disruption of the epithelial layer as described previously (Figure 2, middle panels) [38]. Treatment with azithromycin attenuated the accumulation of inflammatory cells in the lung tissue (Figure 2, right panels). To quantify the accumulation of immune cells in the airways we collected the BAL fluid. In agreement with the histologic appearance of the lung tissue, azithromycin treatment significantly attenuated the accumulation of total BAL immune cells (Figure 3A). Analysis of the BAL leukocyte populations demonstrated that azithromycin treatment significantly decreased the accumulation of macrophages, lymphocytes and neutrophils, with the largest fold-reduction in the number of neutrophils (Figure 3B-D). To exclude the possibility that azithromycin treatment decreased accumulation of immune cells in the airways by a systemic depletion of immune cells, we quantified total and differential leukocytes in the peripheral blood at day 8 post-viral inoculation. We observed no significant differences between PBS and azithromycin-treated cohorts, in terms of total leukocytes (mean cells/μL ± SD, 4100 ± 1300 vs. 4000 ± 600 respectively; p = 0.81) or numbers of macrophages, lymphocytes or neutrophils (data not shown). Thus, azithromycin treatment attenuated lung inflammation in this model of viral bronchiolitis.

### Azithromycin attenuated viral-dependent airway inflammation is associated with decreased concentrations of BAL inflammatory mediators

**Figure 4:**
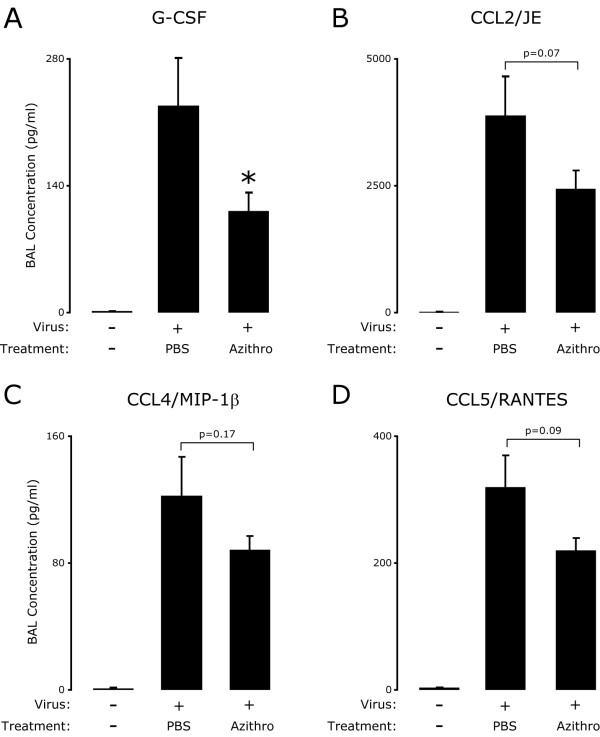
**Azithromycin attenuated viral-dependent airway inflammation is associated with decreased concentrations of BAL inflammatory mediators**. BAL from mice treated as in Figure 1 was analyzed for inflammatory mediators eight days post-inoculation. Concentrations of inflammatory mediators in the cell-free BAL supernatant were determined using a multiplex flow-cytometry based assay (Bio-Rad Laboratories). Groups are labeled as in Figure 2B and values are the mean ± SEM (n = 11, Azithro; n = 12, PBS) of G-CSF (**A**), CCL2/JE (**B**), CCL4/MIP-1β (**C**), CCL5/RANTES (**D**). A significant decrease between PBS and azithromycin treatment is indicated (*, p < 0.05).

Based on the observation that azithromycin treatment decreased immune cell accumulation on day 8 post-inoculation, we proposed that azithromycin treatment would also be associated with decreased concentration of BAL inflammatory mediators. Compared to SeV inoculated mice treated with PBS, treatment with azithromycin attenuated the expression of multiple BAL chemokines and growth factors (Figure 4A-D, column 2 versus 3). Importantly, we observed a significant azithromycin-dependent decrease in G-CSF and decreased concentrations, albeit not statistically significant, of CCL2/JE, CCL3/MIP-1α, CCL4/MIP-1β and CCL5/RANTES; all these are proteins known to mediate viral immune response (e.g., chemotaxis and activation of inflammatory cells at site of inflammation). In addition, azithromycin treatment resulted in trends toward lower concentrations of multiple other inflammatory mediators in the BAL (IL-1β, IL-5, IL-6, IL-9, IL-10, IL-12, GM-CSF and IFN-γ), but had no effect on concentration of IL-17 and CXCL1/KC (data not shown). These data demonstrate that azithromycin moderated the viral-dependent secretion of multiple BAL mediators, several of which are critical for chemotaxis and activation of inflammatory cells.

### Azithromycin modulation of viral-dependent airway inflammation is independent of Sendai viral load

**Figure 5:**
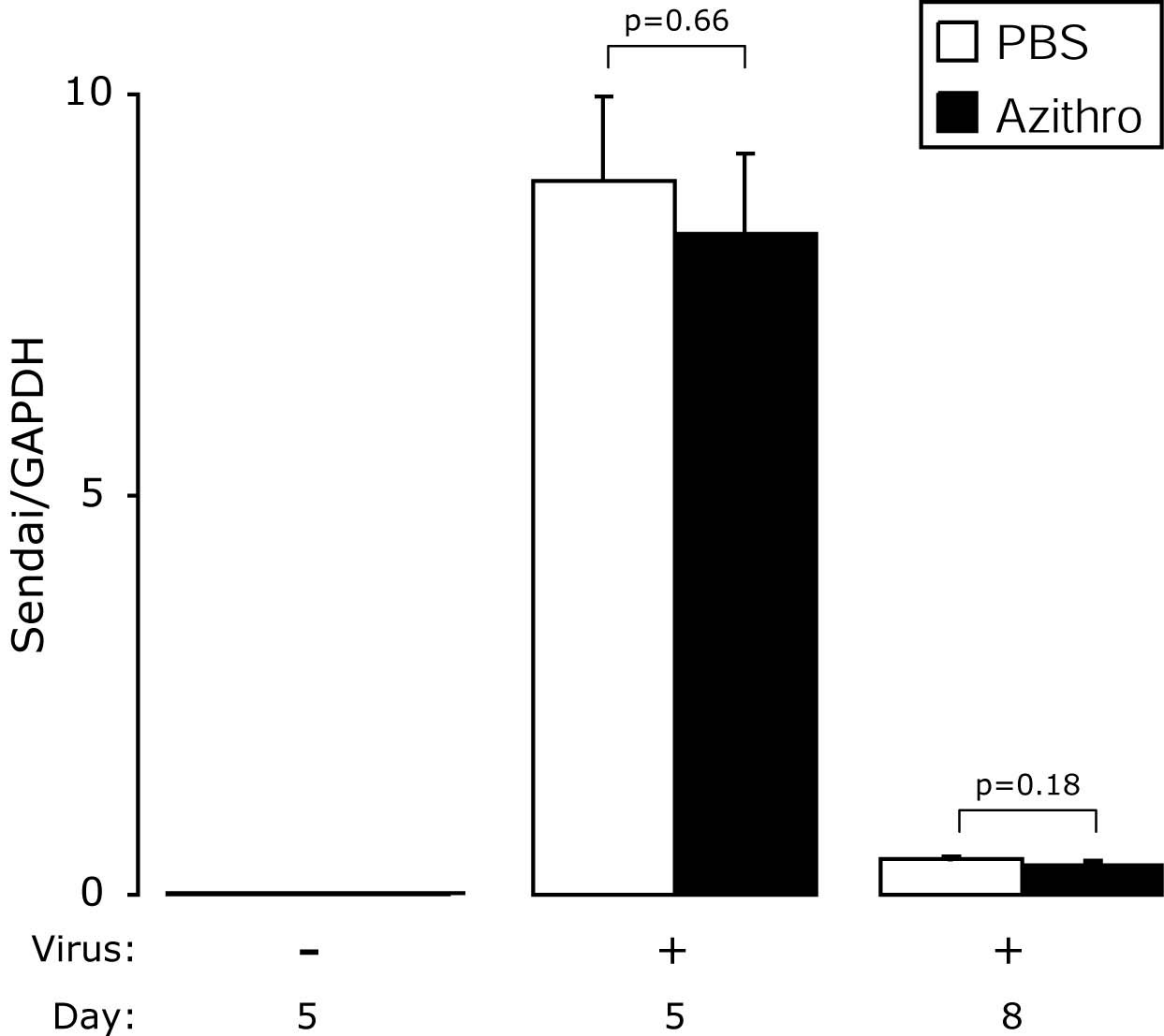
**Azithromycin attenuated viral-dependent airway inflammation is independent of Sendai virus load**. Mice were inoculated with SeV and treated as in Figure 1. Five and eight days post-inoculation, whole-lung RNA was analyzed for Sendai virus-specific and GAPDH RNA by one-step fluorogenic reverse transcriptase-polymerase chain reaction (RT-PCR). The mean of duplicate measurements of SeV-specific RNA was normalized to GAPDH and reported as the SeV to GAPDH ratio. Values are the mean ± SEM (n = 6, day 5; n = 4, day 8).

To investigate whether azithromycin had an effect on SeV burden in the lung tissue, we quantified SeV-specific RNA at day 5 and 8 post-viral inoculation. These time points were chosen based on our previous data that demonstrated peak viral load in the lungs occurred on day 5 post-inoculation, and virus clearance on day 8 post-inoculation [31]. There were no differences in SeV-specific RNA between PBS or azithromycin treated mice suggesting azithromycin does not directly alter viral replication or clearance (Figure 5). Thus, in this model of viral bronchiolitis, azithromycin anti-inflammatory properties are independent of an anti-viral property.

### Azithromycin treatment attenuated chronic viral-dependent airway inflammation

**Figure 6:**
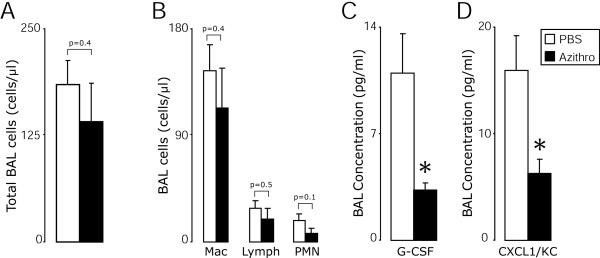
**Azithromycin treatment attenuated chronic viral-dependent airway inflammation**. Mice were inoculated with SeV and treated as in Figure 1. Twenty-one days post-inoculation, lung sections and BAL fluid were harvested. Values are the mean ± SEM (n = 10, Azithro; n = 8, PBS) of total BAL cells (**A**), macrophages, lymphocytes and neutrophils in the BAL (**B**), and concentrations of the chemokines: G-CSF (**C**) and CCL1/KC (**D**). A significant decrease between PBS and azithromycin treated mice is indicated (*, p < 0.05).

As noted above, azithromycin treatment from day 0 through day 7 post-viral inoculation attenuated viral-dependent airway inflammation at day 8 (peak of inflammation). Next we investigated whether azithromycin would also modify the chronic inflammatory phase of the infection. On day 21 post-viral inoculation, the accumulation of total BAL immune cells was elevated compared to naive mice. There were fewer total cells (although not statistically significant) in the BAL of the azithromycin treated cohort compared to those treated with PBS (Figure 6A). There was a trend toward fewer BAL neutrophils in the azithromycin treated mice (Figure 6B). Moreover, azithromycin treatment resulted in a significant decrease in the BAL concentrations of G-CSF and CXCL1/KC (Figure 6C-D), and in a trend toward a decreased concentration of CCL2/JE (data not shown). No statistical difference was noted between the azithromycin and PBS treated mice in terms of the extent of mucous cell metaplasia (PAS score 1.1 vs. 1.0 respectively; p = 0.58). These results demonstrated that azithromycin treatment altered not only acute airway inflammation, but modified certain key aspects of the chronic inflammatory phase of the infection.

## Discussion

This study demonstrated that azithromycin possessed beneficial anti-inflammatory properties in a mouse model of paramyxoviral bronchiolitis. Azithromycin treatment improved the course of acute disease, evidenced by decreased weight loss and attenuated accumulation of BAL inflammatory cells and chemokines. Although not statistically significant, we noted a trend toward lower mortality in the azithromycin treated mice. We also observed that early azithromycin treatment was associated with modulation of certain features of the chronic post-viral inflammatory phase. To the best of our knowledge, this is the first study to demonstrate the beneficial effects of azithromycin in a mouse model of viral bronchiolitis and suggests this drug may also have beneficial effects in human bronchiolitis.

Our results are in agreement with a previous study by Sato and colleagues, who investigated the effect of erythromycin treatment using an in vivo model of influenza pneumonia [39]. That study showed that erythromycin treatment in mice resulted in improved survival, decreased weight loss, and attenuated airway inflammation. Our results extend those findings by demonstrating that a clinically better tolerated macrolide displayed anti-inflammatory properties in a different viral infection model (i.e., a parainfluenza viral bronchiolitis vs. influenza pneumonia). In addition, we demonstrated that treatment of the acute inflammation is associated with attenuation of the chronic post-viral inflammatory phase.

Our results revealed that azithromycin had no effect on SeV viral kinetics in the lung tissue at day 5 and 8 post-viral inoculation, time points that corresponded to the peak of viral load in the lungs and virus clearance respectively [31]. Accordingly, in this case we conclude that azithromycin has anti-inflammatory, but no direct in vivo anti-viral properties. This observation agrees with the previously mentioned report, in which erythromycin treatment did not alter influenza viral kinetics [39]. Although weight loss is attenuated and viral clearance is not compromised by treatment with azithromycin, it remains unclear how azithromycin or other macrolides would alter additional clinical outcomes of a human viral infection such as nasal congestion, nasal discharge, and cough as we have not developed techniques to quantitate these in the mouse. We do note that a recent study found that macrolide antibiotics inhibited RSV infection in isolated human tracheal epithelial cells [40]. These apparently conflicting results could be related to differences between in vivo and in vitro viral infection models, use of different paramyxoviruses, or to different dosing regimens and pharmacologic properties of the drugs.

During the peak inflammatory response, azithromycin treatment attenuated cellular influx in the lung tissue and BAL. Decreased cellular influx in site of inflammation is consistent with previous reports in other inflammatory models in which macrolides attenuated neutrophilic inflammation induced by inhaled LPS [41,42] or intratracheal *P. aeruginosa *infection [43]. The effect of azithromycin, in our viral bronchiolitis model, does not appear to be neutrophil-specific since the accumulation of macrophages and lymphocytes were also attenuated. In previous work, we demonstrated that azithromycin had the most profound effect on eosinophils in an in vivo allergic model of airway inflammation [30]. Taken together, these observations suggest that macrolides possess broad anti-inflammatory properties that can attenuate the accumulation of multiple cell types in various airway inflammatory models.

One limitation of this study is the initiation of azithromycin treatment on the day of infection. Another limitation is that we did not test for a beneficial treatment effect of azithromycin on other respiratory viruses, such as RSV. RSV is a human pathogen and in our experience RSV infection of mice results in pneumonia rather then bronchiolitis. We have found that SeV replicates at high efficiency in the mouse lung and results in acute inflammation of the small airways (i.e., bronchiolitis) that better mimics human bronchiolitis. Since we have not tested for a beneficial treatment effect of azithromycin on RSV infection it is difficult to compare our results to previous studies that modulated the host immune response by blocking the CX3C chemokine activity of the G protein of RSV [44-46]. Future studies will be required to determine if different treatment regimens, such as initiation of treatment a few days after inoculation or alternate dosing regimens would result in a similar beneficial treatment effect on SeV as well as other respiratory viruses.

Although the precise biochemical mechanisms responsible for the anti-inflammatory effects of macrolides are not defined, this family of drugs can inhibit multiple cellular processes involved in an inflammatory response. For example, macrolides have been shown to inhibit neutrophil chemotaxis, leukocyte-epithelial cell adhesion, cytokine secretion and cytokine-dependent intracellular signaling [43,47]. In this regard, macrolides block NF-κB and AP-1 dependent gene transcription of inflammatory mediators [42,48,49]. Thus, additional studies will be required to further define the precise cellular mechanisms responsible for the anti-inflammatory effects of azithromycin and to determine the optimal dosing regimens required to attenuate both the acute and chronic post-viral inflammatory phenotypes.

Previous clinical studies have shown that macrolides are beneficial in the treatment of inflammatory airway diseases such as diffuse panbronchiolitis [13], cystic fibrosis [14], and asthma [15-25]. Two previous studies investigated the effects of macrolide treatment in children hospitalized with RSV bronchiolitis [26,27]. Tahan et al. [27] revealed that a 21 day course of clarithromycin treatment reduced length of hospital stay, the duration of additional treatments (supplemental oxygen, intravenous fluids and bronchodilators) and the day 21 concentrations of IL-4, IL-8 and eotaxin in the serum. However, this study was limited by a relatively small sample size (n = 21). In a recent larger study, Kneyber et al. found that azithromycin treatment did not improve the early disease course in infants hospitalized with RSV bronchiolitis [26]. This study, although important, had two main limitations that may have obscured any potential benefits of the macrolide. First, the researchers designed an equivalence study based on the assumption that a difference less than ± 49.4 hours (approximately ± 2 days) in length of hospitalization would be considered as equivalence (i.e., no benefit for treatment). Therefore, this study was not powered to detect smaller differences in length of hospitalization. Second, the researchers recruited only 71% of the required study population that was determined based on their power analysis. This early termination of the trial prevents definitive conclusions.

The current study highlights the importance of measuring macrolide-dependent effects during the early phase of the viral infection as well as the late phase. In addition we have identified certain growth factors and chemokines (i.e., G-CSF, CCL2, CCL4, CCL5, and CXCL1) that could be tracked to establish a beneficial treatment effect. Accordingly, when planning a human study we feel early and long-term follow-up of clinical and biochemical endpoints should be included in the study design.

Our study revealed that treatment of mouse SeV bronchiolitis with azithromycin during the acute infection would attenuate acute and chronic airway inflammation, and also decrease the chronic post-viral pathologic abnormalities. However, we do not recommend the off-label use of azithromycin during RSV infection until additional prospective randomized clinical trials support its use since excessive use of macrolides has correlated with increased prevalence of macrolide-resistant organisms such as Streptococcus pneumonia [50].

## Conclusions

Our results extend previous findings obtained in different in vivo models by demonstrating that azithromycin possessed anti-inflammatory properties in an in vivo model of viral bronchiolitis. We found that early treatment during viral infection is associated with attenuation of acute and chronic airway inflammation. Azithromycin treatment improved the course of acute disease, evidenced by decreased weight loss and attenuated accumulation of BAL inflammatory cells and chemokines. Our data revealed that early azithromycin treatment also modulates the chronic post-viral inflammatory phase. These results support the rationale for future prospective randomized clinical trials that will evaluate the effects of macrolides on acute viral bronchiolitis and their long-term consequences.

## Abbreviation list

ANOVA: Analysis of Variance; BAL: Bronchoalveolar Lavage; GAPDH: Glyceraldehyde 3-Phosphate Dehydrogenase; H&E: Hematoxylin and Eosin; PAS: Periodic Acid-Schiff; RSV: Respiratory Syncytial Virus; SeV: Sendai Virus.

## Competing interests disclosures

The authors declare that they have no competing interests.

## Authors' contributions

AB: Designed the study, performed the experiments, performed statistical analysis, interpreted the data, and wrote the manuscript. CLM, SG: Performed the experiments, participated in revision of the manuscript, provided final approval of the manuscript. CLC, SLB: Participated in study design, participated in revision of the manuscript, provided final approval of the manuscript. MJW: Designed the study, performed statistical analysis, interpreted the data, and wrote the manuscript. All authors read and approved the final manuscript.
